# LATE ACUTE REJECTION IN LIVER TRANSPLANT: A SYSTEMATIC REVIEW

**DOI:** 10.1590/S0102-67202015000300017

**Published:** 2015

**Authors:** Lucas Souto NACIF, Rafael Soares PINHEIRO, Rafael Antônio de Arruda PÉCORA, Liliana DUCATTI, Vinicius ROCHA-SANTOS, Wellington ANDRAUS, Luiz Carneiro D'ALBUQUERQUE

**Affiliations:** Liver and Gastrointestinal Transplant Division, Department of Gastroenterology, University of São Paulo, School of Medicine, São Paulo, SP, Brazil

**Keywords:** Transplante de fígado, Rejeição, Revisão sistemática

## Abstract

**Introduction:**

Late acute rejection leads to worse patient and graft survival after liver transplantation.

**Aim:**

To analyze the reported results published in recent years by leading transplant centers in evaluating late acute rejection and update the clinical manifestations, diagnosis and treatment of liver transplantation.

**Method:**

Systematic literature review through Medline-PubMed database with headings related to late acute rejection in articles published until November 2013 was done. Were analyzed demographics, immunosuppression, rejection, infection and graft and patient survival rates.

**Results:**

Late acute rejection in liver transplantation showed poor results mainly regarding patient and graft survival. Almost all of these cohort studies were retrospective and descriptive. The incidence of late acute rejection varied from 7-40% in these studies. Late acute rejection was one cause for graft loss and resulted in different outcomes with worse patient and graft survival after liver transplant. Late acute rejection has been variably defined and may be a cause of chronic rejection with worse prognosis. Late acute rejection occurs during a period in which the goal is to maintain lower immunosuppression after liver transplantation.

**Conclusion:**

The current articles show the importance of late acute rejection. The real benefit is based on early diagnosis and adequate treatment at the onset until late follow up after liver transplantation.

## RESUMO

**Introdução:**

A rejeição aguda tardia apresenta resultados com pior sobrevida do paciente e do enxerto após o transplante de fígado.

**Objetivo:**

Analisar os resultados publicados na literatura nos últimos anos pelos principais centros de transplante sobre o tema rejeição aguda tardia para *a* tualização analisando suas manifestações clínicas, diagnóstico e tratamento.

**Método:**

Foi realizado uma revisão sistemática da literatura, utilizando o banco de dados PubMed/Medline com os descritores relacionados à rejeição aguda tardia nos artigos publicados até novembro de 2013. Foram analisados dados demográficos, imunossupressão, rejeição, infecção, bem como as taxas de sobrevida do enxerto e do paciente.

**Resultados:**

A rejeição aguda tardia no pós transplante de fígado mostra pior resultado na sobrevida do enxerto e do paciente. A grande maioria dos estudos foram coortes retrospectivas e descritivas. A incidência de rejeição aguda tardia variou de 7-40% a partir destes estudos. A rejeição aguda tardia é uma das causas de perda do enxerto. Rejeição aguda tardia tem sua definição variável definida em relação ao tempo. Sua evolução apresenta resultado diferente em relação à sobrevida do enxerto, podendo evoluir para rejeição crônica, apresentando pior prognóstico. A rejeição aguda tardia está presente no momento em que se tende a manter menor imunossupressão, alguns meses depois transplante.

**Conclusão:**

Os artigos atuais mostram a importância da rejeição aguda tardia. O benefício real está no diagnóstico precoce e no tratamento adequado durante o episódio e no seguimento tardio após transplante de fígado.

## INTRODUCTION

Acute cellular rejection has been a common cause of graft loss and an indication for re-transplantation. Advances in immunosuppression have improved the outcome of transplantation 7 . However, late acute rejection appears to result in a different outcome with worse patient and graft survival after liver transplantation 7 9 .

Late acute rejection has been variably defined as ocurring more than one, three, or six months after transplantation. Therefore, it differs from early acute cellular rejection, which occurs less than three months after liver transplantation 7 9 . The focus of histologic findings may be portal, central, or both, but the central component is more frequently, and is often associated with poor compliance and is more often refractory to treatment 7 . Late acute rejection causes graft loss, decreased patient survival, chronic rejection and worse prognosis. Late acute rejection occurs during a period in which immunosuppression is lower months after liver transplantation 1 6 7 9 . 

It is important to pay more attention to this important clinical entity, which is associated with graft loss and patient death. The aim of this study was to systematically review the literature on late acute rejection. 

## METHODS

Systematic review was performed using electronic search in Medline-PubMed database in English. The search was completed in November 2013. 

The research questions and the inclusion and exclusion criteria were developed using the PICO method, which includes data on patients, interventions, comparison classes or controls, outcome structures and inclusion/exclusion criteria. The interventions considered were those performed after liver transplantation with late acute rejection.

**FIGURE 1:**
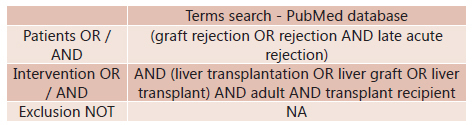
Terms search on PubMed database using PICO structure

The terms for each group were sought in combination using the ''OR'' operator. The results for the search terms, which formed the ''P'' (Patients) group were combined with the result for searches that formed the ''I'' (Intervention) group using the ''AND'' operator and subsequently with the ''exclusion keywords'' using the ''NOT'' operator ( Figure 1 ).

The Medline search was performed through PubMed (www.ncbi.nlm.nih.gov/pubmed) and was adapted using the Mesh-terms (graft rejection OR rejection) AND (liver transplantation OR liver graft OR liver transplant) AND adult AND transplant recipient. After this initial search was realized, other selections with more specific terms and mesh terms using (rejection OR graft rejection AND late acute rejection) AND (liver transplantation OR liver graft OR liver transplant) were performed.

**TABLE 1:** Overall analysis of the studies on late acute rejection

Studies	Type /Time	LAR definition (histologically)	Incidence /Factor	outcomes
Wang, G.Y. et al. 2013 10	Retrospective; 40 biopsies performed on 37 patients	Six months after LT	ACR (n=24) Relative eosinophil count was higher than non-ACR	>blood eosinophil count was a valuable biomarker for predicting LAR after LT
Uemura, T. et al. 2008 9	Retrospective; 1604 adult LT; from 1985 to 2003.	> Six months after LT	19.0% (305 /1604)	Patient (p=0.0083) and graft survival (p=0.0075) were significantly lower in the LAR
Ramji, A. et al. 2002 6	Retrospective; 524 LT performed from 1989 to 2000.	Six months after LT	23% (97/415); median 402 days post LT (range, 180 to 3137 days)	> CR in patients developed LAR (p= 0.04) 79% mild 5% ST resistant
Thurairajah, P.H. et al. 2013 7	Retrospective; 970 adult LT from 2000 to 2010.	Three months after LT	11% (103/970), mean time of 565 days (median, 311 days; range, 90-2922 days)	Graft survival (10 years) was 74% in LAR vs 81% in those without AR (p=0.01)
Akamatsu, N. et al. 2006 1	247 adult LDLT from January 1996 to March 2005.	> Six months after LDLT	7% (15 cases) Median time 302 day (range:182-1490)	Survival based on immunosuppression: tacrolimus (n=166) vs cyclosporine (n =38) (p< 0.0001)
Florman, S. et al. 2004 2	Total of 532 recipients; more than 1000 days follow-up	33 months after LT	8,1% (43) mean time 1545 ± 441 d post-LT. 38 of the 43 (88.4%) patients with LAR had EAR episodes before 1000 days post-LT vs. only 295 of the 488 patients (60.5%) that did not have LAR (p< 0.01)	Overall patient survival for LAR (n=43) is 81.4% vs 82.0% without LAR (n=488) (p =ns).
Junge, G. et al. 2005 3	1426 LT performed from 1988 till April 2002.	> Three months after LT	AR in 5% (52) among 47 patients. LAR 79% demonstrated previous EAR	CR was 3.7%. No significant difference in patient survival (with or without LAR)
Neil, D.A.H. et al. 2001 5	Prospective; evaluated the delay on diagnoses of Bx	> One month post LT	40.7% (11) LAR. Incidence in LAR is much greater at 25%.	No difference in severity and RAI p>0.05 (EAR vs LAR); worse prognosis of LAR
Wiesner, R.H. et al. 2006 11	9646 adult LT from June 1995 to April 2004	≥ Six months post- LT	LAR independent risk factor for late graft loss (HR=1.99, p<0.001) and for late death (HR=1.98, p=0.001)	MMF with TAC and ST decreased risk of LAR, in patients with HCV, HBV and nonviral disease

LAR=late acute rejection; AR=acute rejection; EAR=early acute rejection; LT=liver transplantation; LDLT=liver donor liver transplantation; ST=steroid; Bx=biopsy proven acute cellular rejection scored in Banff classification; CR=chronic rejection; RAI=rejection activity index; TAC=tacrolimus; MMF=mofetil

The studies were analyzed by two independent researchers (LSN and RAP), and a consensus meeting was held to reach a final decision. The study was approved by a Research Ethics Committee.

## RESULTS

**FIGURE 2:**
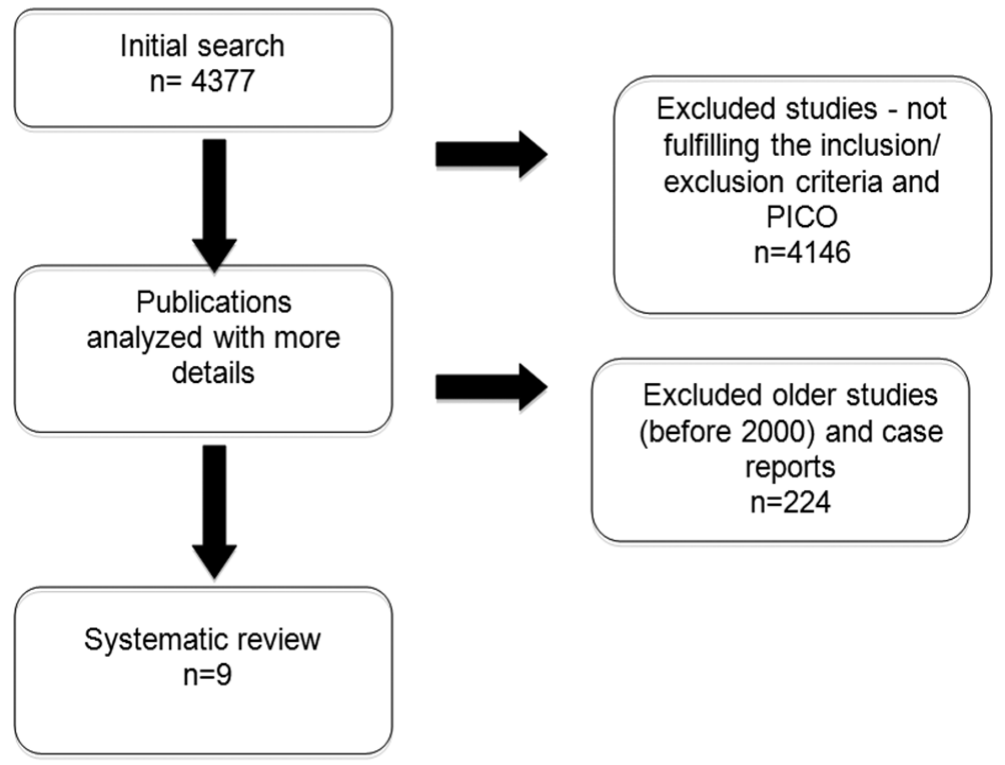
Diagram of this systematic review showing the steps for articles selection

This systematic review initially showed 4377 articles, and after a specific search, 234 articles were selected ( Figure 2 ). Of these, 20 studies were selected according to inclusion criteria and nine were selected for this review.

In this review, was noted the importance of late acute rejection in the post-liver transplant, mainly affecting patient and graft survival. Table 2 shows the overall analysis of the nine selected studies. The definition of late acute rejection is more than six months in most studies; however, the diagnosis of early acute rejection occurs within the first month after liver transplant, and that of late acute rejection occurs after three months.

Almost all of these cohort studies were retrospective and descriptive. The incidence of late acute rejection was 7-40% in these studies. Only one related incidence was greater at 25%. Other significant findings were the significantly lower patient and graft survival. The evolution to chronic rejection is higher in patients who develop late acute rejection ( Table 2 ).

The immunosuppression regimen after liver transplantation and the therapeutics used during episodes of late acute rejection are shown in Table 2 . The majority of papers reported therapy with steroid and tacrolimus with strict therapeutic drug monitoring after liver transplantation. More than six months after the surgery, tacrolimus or cyclosporine were maintained at 5 to 10 μg/l and 100 to 150 μg/l, respectively. During the episode, an intravenous bolus of 1 g of methylprednisolone was used daily for two days followed by oral prednisolone. Table 3 shows that some cases with steroid-resistance, persistent acute rejection or renal insufficiency have options to improve the results.

## DISCUSSION

The importance of this study involved early diagnosis and successful treatment, both of which are essential for improving the prognosis and survival rate of the graft and the patient 5 7 . In the reviewed literature, was observed a small number of specific studies in this area, with most of the articles being retrospective and descriptive cohort.

Late acute rejection occurred primarily with an incidence rate of 7-23% 1 7 6 . Only one study reported an incidence of 41% 5 . The definition for the time period for late acute rejection is obscure. There is no consensus on the time period, and most of the studies used 3-6 months 6 9 . One used more than one year^2^ but did not show a survival difference between late and early acute rejection. Another used one month as the beginning of the time period for late acute rejection 5 . A long-term retrospective study 9 showed incidence of 19% and significantly lower patient and graft survival 9 . Other long-term papers showed similar results, with an incidence rate of 11% and lower survival (10 year follow up) with late acute rejection than without acute rejection 7 .

Wang et al. 10 performed 40 biopsies and demonstrated an increase in blood eosinophil count as a valuable biomarker for predicting late acute rejection after liver transplantation 10 . Neil et al. 5 presented data on early diagnosis that evaluated the delay in diagnosis based on biopsy and reported no difference between early and late acute rejection in the severity and rejection acute index (RAI) 5 .

Regarding the use of an immunosuppressive regimen, therapy may affect survival. Tacrolimus and cyclosporine showed a significant difference and better results in the tacrolimus group 1 6 . Another important finding is that mycophenolate mofetil with tacrolimus and steroid decreased the risk of late acute rejection in patients with hepatitis virus C, hepatitis virus B and no viral disease 8 11 .

**TABLE 2:** Immunosuppression regimen and therapeutics during episodes of late acute rejection

Studies	Immunosuppression regimen	Therapeutic LAR episode
Ramji, A. et al. 2002^6^	32 patients (33%) were ST tapered within the previous 8 weeks, 15 patients (16%) were not on any ST, 48 (50%) had ST dose of prednisone 5 mg or less daily. 17 patients (18%) had sub therapeutic CsA or TAC levels at least once during the preceding eight weeks: four in TAC (≤5 ng/mL) and 13 in the CsA group (level ≤100 ng/mL)	73% of LAR episodes were treated with pulse intravenous ST. The remaining rejection episodes were treated with an increase in oral prednisone or a change in calcineurin inhibitor agent (CsA to TAC). 6% of LAR episodes were ST resistant and required OKT3. LAR treated with maintenance cyclosporine compared with tacrolimus, 28% vs 14%, respectively (p=0.006).
Junge, G. et al. 2005^3^	N/A	Corticoid bolus therapy was prescribed in 39 patients (81%). Of all the patients with grade 0.5 rejections, 28% (n=7) received a modification of their immunosuppression. AR higher than grade 1 was treated with ST bolus therapy (11%) or a modification of their immunosuppression (30%).
Florman, S. et al. 2004^2^	CsA target levels (ng/dL) were routinely maintained post-LT between 300 and 400 the first month, 250 and 350 the second and third months, 200 and 300 between months 3 and 12, and 100 and 200 after 1 year. TAC target levels (ng/dL) were routinely maintained post-LT between 15 and 20 the first month, 10 and 15 between the second and third months, approximately 10 between 3 and 12 months, and between 5 and 10 after 1 year.	Intravenous ST boluses ± intravenous ST recycle; Over five days (50 mg, then 40 mg, then 30 mg, then 20 mg, then 20 mg, then changed to 20 mg daily orally) for this initial LAR. OKT3 in few cases.
Uemura, T. et al. 2008 ^9^	TAC or CsA with ST. Renal dysfunction or other calcineurin toxicity received azathioprine at 1-2 mg/kg/d (1984-1994) or MMF at 0.5-2 g/d (1995-2004). ST taper was used in all patients. Induction therapy with OKT3 was used in only patients with pre-existing renal failure at the time of transplant. CsA target levels (ng/mL) were routinely maintained post-LT between 250 and 350 in the first month and tapered down to 100 and 200 after one year.	Intravenous bolus of 1 g of methylprednisolone daily for two days followed by recycles of prednisolone. If clinical and histological evidence of persistent acute rejection remained, OKT3 or thymoglobulin was administered intravenously for a total of seven to 14 day followed by a liver biopsy.
Thurairajah, P.H. et al. 2013 ^7^	24 patients (24%) were on monotherapy with a calcineurin inhibitor (21 on TAC and 3 on CsA), 56 patients (57%) were on two immunosuppressors, with the most common combination consisting of an antimetabolite and a CNI (19 azathioprine and TAC, 16 TAC and MMF), 9 patients were on prednisolone and TAC and 18 patients (18%) were on a triple-therapy regimen of CNI, antimetabolites, and corticosteroids.	Pulsed high-dose corticosteroids prednisolone 200 mg/day for three days.
Akamatsu, N. et al. 2006 ^1^	ST and TAC strictly controlled with therapeutic drug monitoring. More than 6 months after LDLT, TAC and CsA were maintained at 5 to 10 μg/L and 100 to 150 μg/L, respectively.	High-dose methylprednisolone (20 mg/kg per day) followed by recycling. Patients with steroid-resistant cellular rejection were treated with MMF and OKT3.

CsA=cyclosporine; OKT3=anti-T-cell monoclonal antibody; MMF=mycophenolate mofetil; LAR=late acute rejection; AR=acute rejection; EAR=early acute rejection; LT=liver transplantation; LDLT=liver donor liver transplantation; ST=steroid; Bx=biopsy; CR=chronic rejection; TAC=tacrolimus; N/A=not applicable

The immunosuppression therapy was steroid and tacrolimus strictly controlled with therapeutic drug monitoring. More than six months after liver transplantation, tacrolimus and cyclosporine were maintained at 5 to 10 μg/l and 100 to 150 μg/l, respectively 1 .

 An episode of late acute rejection should be treated with a bolus of corticosteroids, which was prescribed for 81% 3 and 73% 6 of patients. In addition to intravenous steroid boluses, patients can be treated with steroid recycle^2^ and oral steroids 9 and pulsed high-dose prednisolone at 200 mg/day for three days 7 . High-dose methylprednisolone (20 mg/kg per day) followed by recycling is another option 1 .

Modifications of the immunosuppression (Cyclosporine to Tacrolimus) can be used for different types of rejection. Higher acute rejection than grade 1 was treated with steroid bolus therapy (11%) or a modification of the immunosuppression (30%) 3 . In 6% of late acute rejection episodes, steroid-resistance was encountered and required treatment with anti-T-cell monoclonal antibody (OKT3) 6 in a few cases 2 6 . Patients with steroid-resistant cellular rejection were treated with mycophenolate mofetil and anti-T-cell monoclonal antibody 1 . If clinical and histological evidence of persistent acute rejection remained, OKT3 or Thymoglobulin was administered intravenously for a total of 7 to 14 days 9 . 

Nacif et al. 4 analyzed the blood samples over time 30 days after the liver transplantation and showed a significant correlation between the Tacrolimus blood level and the deterioration of glomerular filtration rate and serum creatinine. Patients with infections had a higher serum level of Tacrolimus. The dose and presence of rejection were significantly different. Blood Tacrolimus levels greater than 10 ng/ml were associated with impaired renal function. Doses greater than 0.15 mg/kg/day were associated with the prevention of acute cellular rejection but predisposed patients to infectious disease 4 .

Patients with renal dysfunction or other calcineurin toxicity received azathioprine at 1-2 mg/kg/d or mycophenolate mofetil at 0.5-2 g/d, and a steroid taper was used in all patients. Induction therapy with OKT3 was used in only patients with pre-existing renal failure at the time of liver transplant. Cyclosporine target levels (ng/ml) were routinely maintained post liver transplantation between 250 and 350 during the first month and tapered down to 100 and 200 after one year 9 .

## CONCLUSION

The current articles show the importance of late acute rejection. In addition, the data support the benefit of early diagnosis and the appropriate treatment of the episode and maintenance therapy during the late period after the liver transplantation.
